# Comparative anatomy of the thoracic muscles of bees (Hymenoptera: Apoidea)

**DOI:** 10.7717/peerj.20532

**Published:** 2026-01-06

**Authors:** Odair M. Meira, Eduardo A.B. Almeida

**Affiliations:** Departamento de Biologia, Universidade de São Paulo, Ribeirão Preto, São Paulo, Brazil

**Keywords:** Apidae, Flight, Mesosoma, Morphology, Systematics

## Abstract

Bees exhibit a remarkable anatomical diversity, with phenotypic traits that reflect broad evolutionary patterns and specific adaptations. Understanding these patterns requires examining key anatomical features, such as thoracic musculature, which drives morpho-functional variation and underscores their extensive phenotypic diversity. The thorax (or ‘mesosoma,’ as it can be referred to in the context of bees and other apocritan Hymenoptera) serves as a power core, housing muscles responsible for leg, wing, and also head and metasomal articulation movements. Despite the role of the thoracic musculature in the flight mechanics of bees, detailed studies are limited to accounts of individual species or small subsets of muscles, with truly comparative analyses being scarce, leaving gaps in understanding muscular variation and phylogenetic significance. To address this, we conducted detailed dissections of 13 species, representing six bee families (Andrenidae, Apidae, Colletidae, Halictidae, Megachilidae, and Melittidae) and three additional apoid wasp taxa (Bembicidae, Crabronidae, Philanthidae), selected to capture a broad range of morphological and phylogenetic diversity. Our results revealed high conservation in mesosomal musculature, with only 16 of 58 muscle groups showing significant variation, primarily in origin points, suggesting a balance between functional constraints and evolutionary flexibility in muscle attachment. Phylogenetically relevant changes were investigated by coding 17 morphological characters, revealing potential synapomorphies for bees or certain lineages. These include the dorsomedial origin of Idlm1 (M. prophragma-occipitalis) in Meliponini, as evident in species such as *Melipona quadrifasciata* and *Tetragonisca fiebrigi*, suggesting a shared derived trait for this tribe. Additionally, the extended origin of IIIscm2 is observed in Andrenidae, Colletidae, and Halictidae, indicating closer evolutionary relationships among these families. Bee-specific modifications, including the non-separation of IItpm7b and IItpm7c by the mesepisternal ridge, distinguished bees from most apoid wasps, interpreted here as a potential synapomorphy for bees. Additional variations, such as the ventral origin of Ivlm3 in select lineages and the branched morphology of IIpcm4, suggest independent evolutionary shifts potentially linked to biomechanical demands. These findings underscore the evolutionary stability and phylogenetic value of bee mesosomal musculature, revealing a conserved framework punctuated by lineage-specific adaptations that may correlate with ecological traits.

## Introduction

Like other arthropods, bees have their bodies covered by a chitinous exoskeleton featuring articulated parts. These parts are moved internally by specialized muscles with well-defined attachment points (Snodgrass, 1927; Snodgrass, 1935). The roles played by these muscles, in coordination with their cuticular attachment points, vary across body regions (Snodgrass, 1935). This variation reflects regional specialization that makes each tagma suited for specific tasks, with differing degrees of musculature specialization. The prominent thorax of a bee serves as a power core and the primary tagma for locomotion. It has two pairs of wings and three pairs of legs attached to it, as is also the case in other flying insects (Snodgrass, 1927; Snodgrass, 1935; Matsuda, 1970). The thoracic muscles are essential for meeting the demands of flight (Dudley, 2000; Dickinson, 2006; Iwamoto, 2011). However, other body parts like the abdomen and head also help with overall movement by maintaining balance during flight. In apocritan Hymenoptera, such specialization is especially pronounced: the enlargement of the mesothorax correlates with the activity of indirect flight muscles, which are essential for rapid wing beats (Chapman, 2013). In these insects, the mesosoma corresponds to the thorax fused with the first abdominal segment, and supports complex locomotor adaptations (Vilhelmsen, Mikó & Krogmann, 2010). The terms “thorax” and “mesosoma” are used interchangeably in this work for simplicity, as the mesosoma can be morphofunctionally interpreted as an expanded thorax.

Classical morphological techniques, especially detailed dissections, remain essential for studying the complex skeletomuscular system of insects. These techniques help document the muscle structure. In this context, “The skeleto-muscular mechanisms of the honey bee” (Snodgrass, 1942) stands out as a landmark, offering unmatched direct access to muscle attachment points, fiber orientation, and tissue features. Detailed studies have improved the identification of homologous structures and have been crucial in documenting the morphological diversity across bee taxa and other groups of Hymenoptera (*e.g.*, Snodgrass, 1942; Wille, 1956; Daly, 1964; Matsuda, 1970; Mikó et al., 2007; Vilhelmsen, Mikó & Krogmann, 2010; Porto, Almeida & Vilhelmsen, 2017; Meira & Gonçalves, 2021). Recently, traditional methods have been supplemented by modern imaging techniques, such as micro-computed tomography (Micro-CT), which provides non-invasive, high-resolution three-dimensional reconstructions of internal anatomy (*e.g.*, Friedrich & Beutel, 2008; Friedrich et al., 2014; Willsch et al., 2020; Aibekova et al., 2022; Aibekova et al., 2025; Meira et al., 2024). Micro-CT allows visualization of delicate internal structures without destructive preparation, preserving the specimen for further study. It enables the observation of muscles and skeletal elements in their natural positions, making it easier to perform 3D and spatial analyses that are challenging to achieve with dissection alone. However, its main limitations include the low contrast of soft tissues without staining and the high cost and computational resources required for data acquisition and processing. The synergy between these approaches, where traditional dissections reinforce and improve the reliability of modern imaging, creates a strong knowledge base that supports comparative research, allowing systematic documentation of anatomical variation across bee lineages and aiding inferences about their functional and evolutionary significance.

The investigation of the bee mesosomal skeletomusculature from a comparative perspective to highlight anatomical variation in this complex is limited to a single study. That study examined only 10 muscles (Wille, 1956). There are between 57 (Snodgrass, 1942) and 58 (Meira et al., 2024) muscle groups in a bee mesosoma (intrinsic mesosomal muscles, plus coxal muscles originating in the mesosoma, *i.e.,* extrinsic leg muscles). This means that several dozen muscles remain unstudied from a comparative perspective. Moreover, there are about 21,000 known bee species (Ascher & Pickering, 2020) inhabiting nearly all terrestrial habitats. Bees exhibit an impressive size range (Messer, 1984; Michener, 2007) from less than two mm (*e.g.*, some *Euryglossina* (Colletidae: Euryglossinae)) to nearly 40 mm (*e.g.*, *Megachile pluto* (Megachilidae: Megachilini)). Their species diversity is also reflected by a wide variety of shapes, sizes, and life histories (Roubik, 1989; Michener, 2007; Danforth, Minckley & Neff, 2019), resulting from the diversification of bees since the early Cretaceous (Almeida et al., 2023). For instance, while flight adaptation is a key feature of winged insects, many bee species thrive in environments with limited light, such as dense vegetation or high-altitude regions where reduced visibility may constrain flight activity to crepuscular or diurnal patterns with adaptations for low-light navigation, and some have evolved flightlessness (Michener, 2007). Comparing the anatomical variation of thoracic muscles among bees can yield important insights into how these differences relate to their diverse ecological and biological strategies.

Addressing the knowledge gaps regarding species diversity of bees, as well as the variation in the suite of 58 mesosomal muscles, is crucial, as comparative analyses of these muscles could uncover new and unique evolutionary conditions in particular lineages, while also enabling the investigation of adaptations related to flight mechanics or to behaviors associated with nesting and other life history traits. Recent advances in morphological analysis and the terminological standardization efforts by Meira et al. (2024) have helped align bee morphological terminology with broader developments in Hymenoptera, making further research in this area both timely and essential for expanding our understanding of this system.

This study offers the first comprehensive comparative analysis of the mesosomal musculature in bees, describing and quantifying muscle variation across lineages, discussing the variation from a phylogenetic perspective, and highlighting its potential functional and biomechanical significance. By integrating detailed dissections of representatives from all bee families and selected apoid wasps, this work establishes a framework for interpreting muscle homology and variation within a conserved yet evolutionarily dynamic system. This integrative approach, combining classical dissections with comparative morphological analysis across multiple lineages, is unique in its scope and provides a valuable reference for future morphological and phylogenetic studies.

## MATERIALS & METHODS

**Figure 1:**
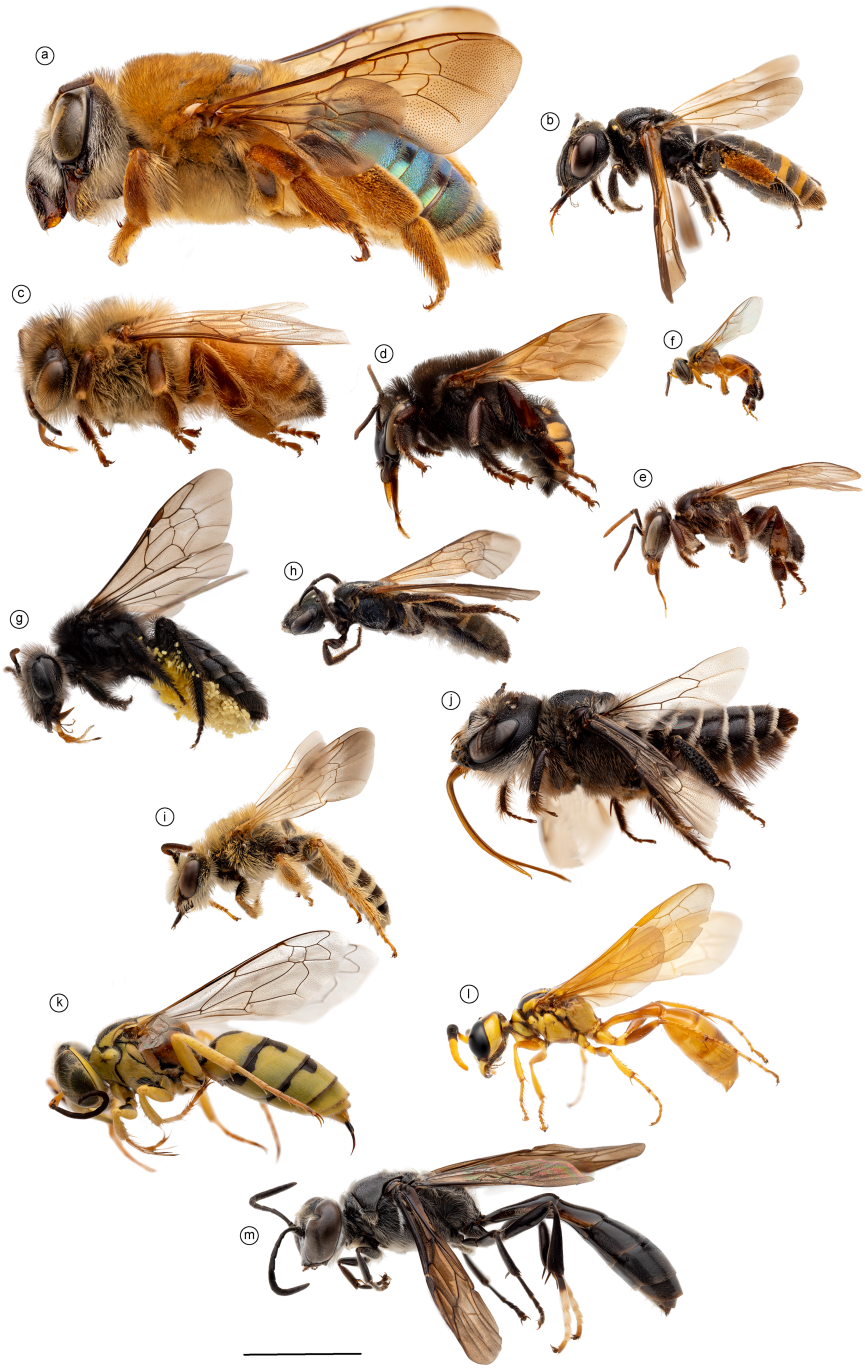
Morphological diversity of Apoidea. Morphological diversity of Apoidea represented by a sample of 13 species analyzed in this comparative research of the mesosomal skeletomuscular morphology. Bees were the main focus of the investigation and were represented by 10 species: (A) *Oxaea flavescens*, female and (B) *Psaenythia bergii*, female [Andrenidae]; (C) *Apis mellifera*, female worker (D) *Melipona quadrifasciata*, female worker, (E) *Schwarziana quadripunctata*, female worker, and (F) *Tetragonisca fiebrigi*, female worker [Apidae]; (G) *Tetraglossula anthracina*, female [Colletidae]; (H) *Oragapostemon divaricatus*, female [Halictidae]; (I) *Hesperapis carinata*, male [Melittidae], (J) *Lithurgus huberi*, female [Megachilidae]; complemented by three apoid wasps: (K) *Steniolia duplicata*, female [Bembicidae], (L) *Trachypus boharti*, female [Philanthidae], and (M) *Trypoxylon lactitarse*, female [Crabronidae]. Scale bar = five mm.

Representative specimens of 10 bee species, representing all major lineages of bees, were selected and sampled to explore the detailed structure of the mesosomal musculature: *Oxaea flavescens* Klug and *Psaenythia bergii* Holmberg [Andrenidae]; *Apis mellifera* Linnaeus, *Melipona quadrifasciata* Lepeletier, *Schwarziana quadripunctata* (Lepeletier), and *Tetragonisca fiebrigi* (Latreille) [Apidae]; *Tetraglossula anthracina* (Michener) [Colletidae]; *Oragapostemon divaricatus* (Vachal) [Halictidae]; *Lithurgus huberi* Ducke [Megachilidae]; and *Hesperapis carinata* Stevens [Melittidae]. This sampling encompasses a considerable range of morphological variation, with body sizes ranging from 4 to 22 mm (Fig. 1), and phylogenetic diversity that covers all the extant bee diversity (according to current hypotheses, *e.g.*, Almeida et al. 2023). Specimens of three apoid wasp species were selected and investigated in detail for comparison with the bee morphology: *Steniolia duplicata* [Bembicidae], *Trypoxylon lactitarse* de Saussure, 1867 [Crabronidae], and *Trachypus boharti* Rubio-Espina 1975 [Philanthidae]. Voucher specimens of the species investigated in this study and the dissected specimens are deposited in the *Coleção Entomológica “Prof. J.M.F.Camargo”* (RPSP), Universidade de São Paulo, Ribeirão Preto, Brazil. For each species, one individual was dissected in detail, and the process involved gradually exposing and photographing each muscle group. The dissection process was repeated with additional specimens when necessary to verify shape differences or confirm uncertain observations.

### Preparation of the specimens

The specimens were killed in a cyanide jar and transferred to a vial containing the fixative Dietrich fixative, a formaldehyde-based solution. These specimens remained in Dietrich for about three days, then preserved in absolute ethanol for long-term storage. Ethanol-preserved specimens were stained with B-I2E (2.5%) for 30 min immediately before dissection to ensure optimal muscle visibility. The specimens were washed in 100% ethanol to remove the remaining excess stain and carried to the dissection step for about 30 min. The B-I_2_E (2,5%) solution is prepared by adding 8.33 mL of I_2_E (15% w/v) to 25 mL of Phosphate buffer and 16.7 mL of bi-distilled water (modified from Dawood et al., 2021) and the I_2_E = alcoholic iodine (15% w/v) solution can be prepared by combining 15 mL of pure I_2_ to 100 mL of absolute ethanol (modified from Li et al., 2016). Staining with 2% iodine was used to facilitate muscle visualization. As the iodine staining is not permanent, the procedure was repeated when necessary.

### Dissection techniques

**Figure 2:**
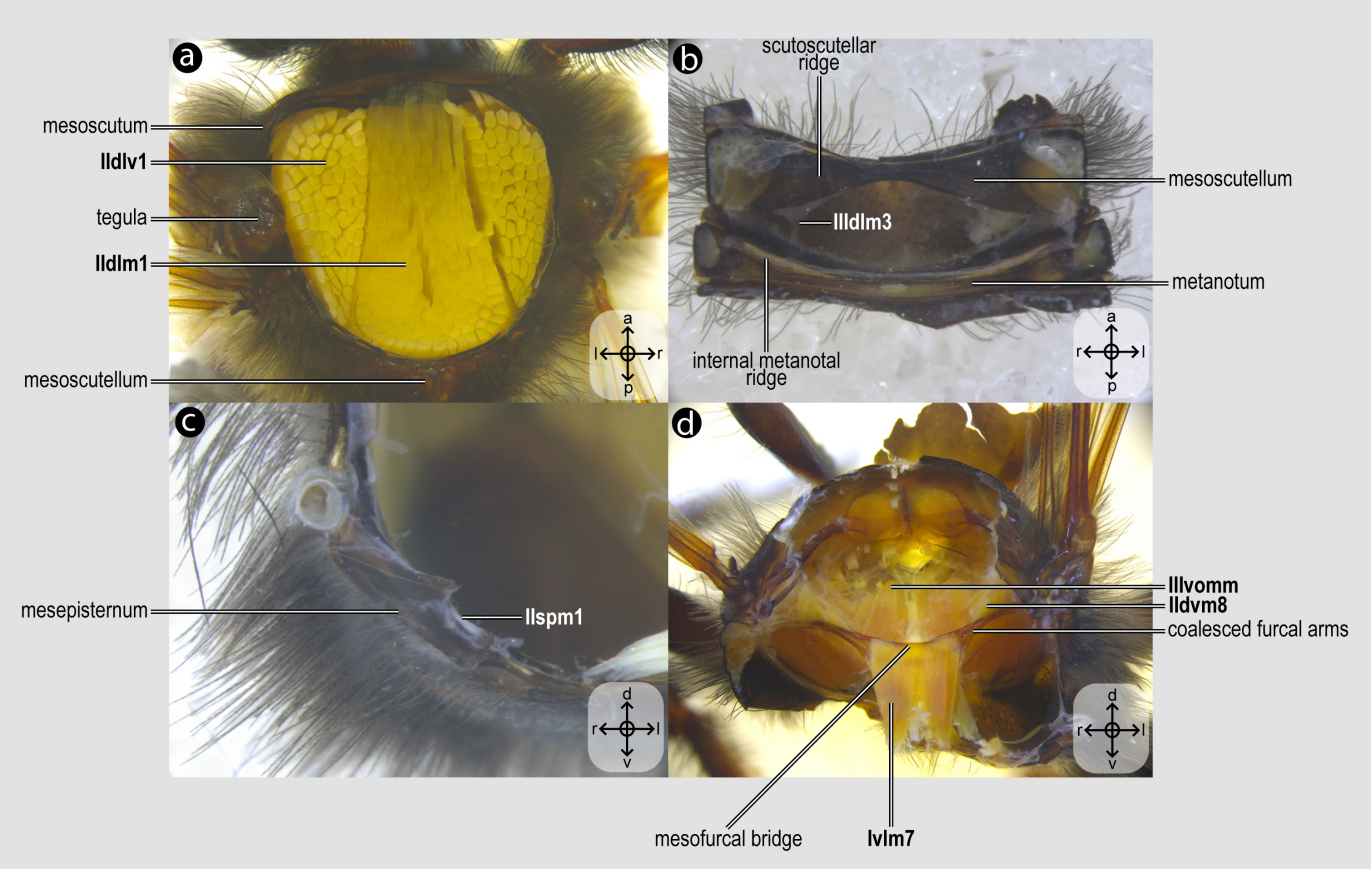
Mesothoracic skeletomusculature. Mesothoracic skeletomusculature of *Melipona quadrifasciata*: (A) indirect flight muscles—dorsal view of mesosoma with mesoscutum removed; (B) scutoscutellar muscles—ventral view of mesoscutellum; (C) anterior thoracic spiracular muscles—anterior view of mesepisternum; (D) muscles associated with the meso/metafurca—anterior view of the meso/metafurca. Muscle labels are displayed in bold and larger font to differentiate them from labels applied to sclerites and body regions; photomicrographs not to scale. Stars indicate structure orientation according to three axes: anterior–posterior, dorsal–ventral, and left–right.

**Figure 3:**
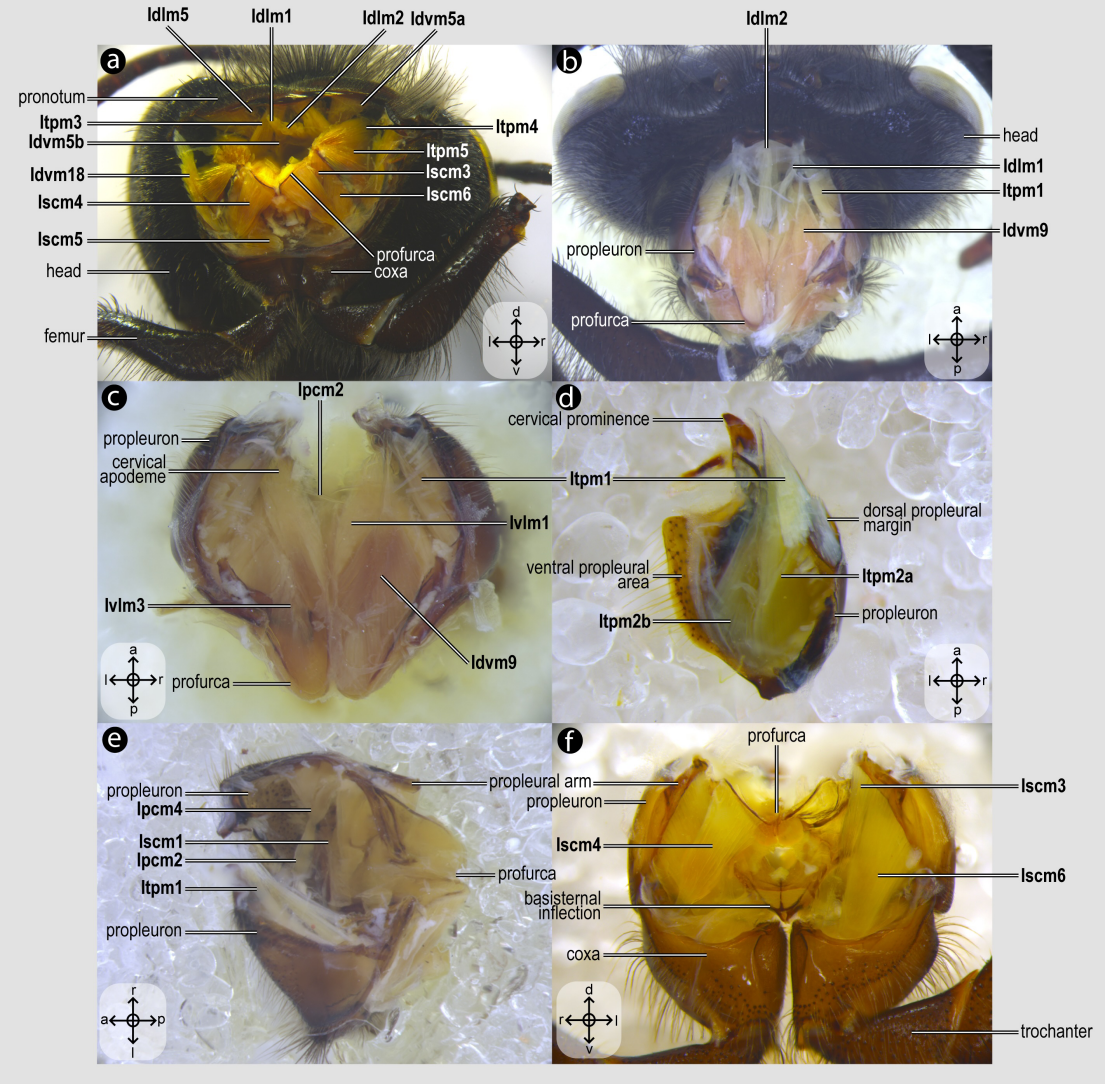
Prothoracic skeletomusculature. Prothoracic skeletomusculature of *Melipona quadrifasciata*: (A) muscles associated with the profurca, pronotum, and propleura—posterolateral view of the head and propectus; (B) muscles associated with the head and propectus—dorsal view of propectus; (C) muscles associated with the propectus—dorsal view of propectus; (D) muscles associated with the propleura—dorsal view of propleuron; (E) muscles associated with propectus—dorsolateral view of propectus; (F) muscles associated with propectus and procoxa—posterior view of propectus. Muscle labels are displayed in bold and larger font to differentiate them from labels applied to sclerites and body regions; photomicrographs not to scale. Stars indicate structure orientation according to three axes: anterior–posterior, dorsal–ventral, and left–right.

**Figure 4:**
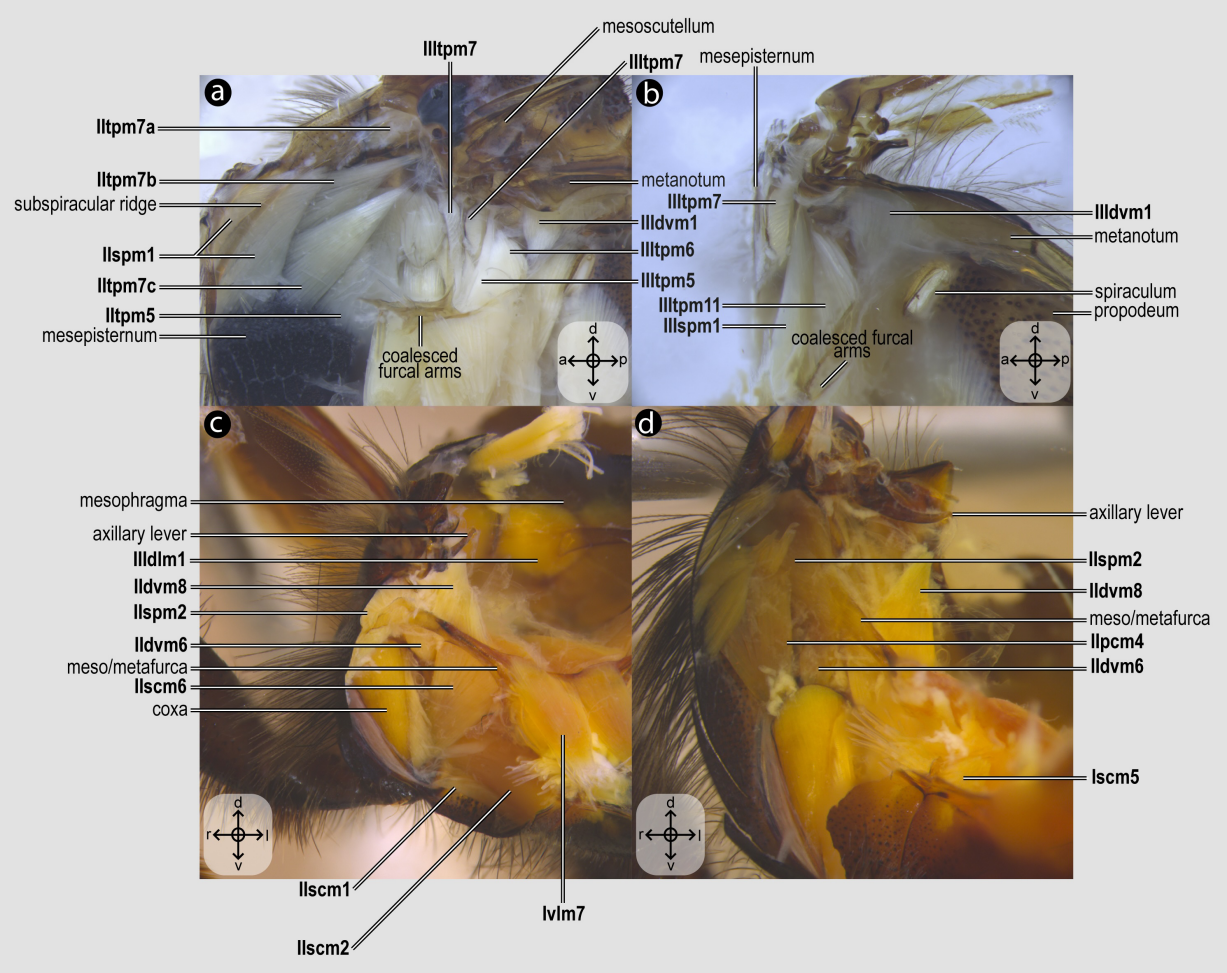
Direct flight muscles and mesocoxal skeletomusculature. Direct flight muscles and mesocoxal skeletomusculature of *Melipona quadrifasciata*: (A) muscles associated with the mesoaxillary sclerites—mesal view of the mesepisternum; (B) muscles associated with the metaaxillary sclerites—mesal view of the metapectus; (C) mesocoxal muscles associated with the meso/metafurca—anterior view of the meso/metafurca; (D) pleural muscles associated with the meso/metafurca—anterior view of the meso/metafurca. Muscle labels are displayed in bold and larger font to differentiate them from labels applied to sclerites and body regions; photomicrographs not to scale. The star depicted in (A) indicates structure orientation according to three axes: anterior–posterior, dorsal–ventral, and left–right. All figures oriented according to the star depicted in (A).

**Figure 5:**
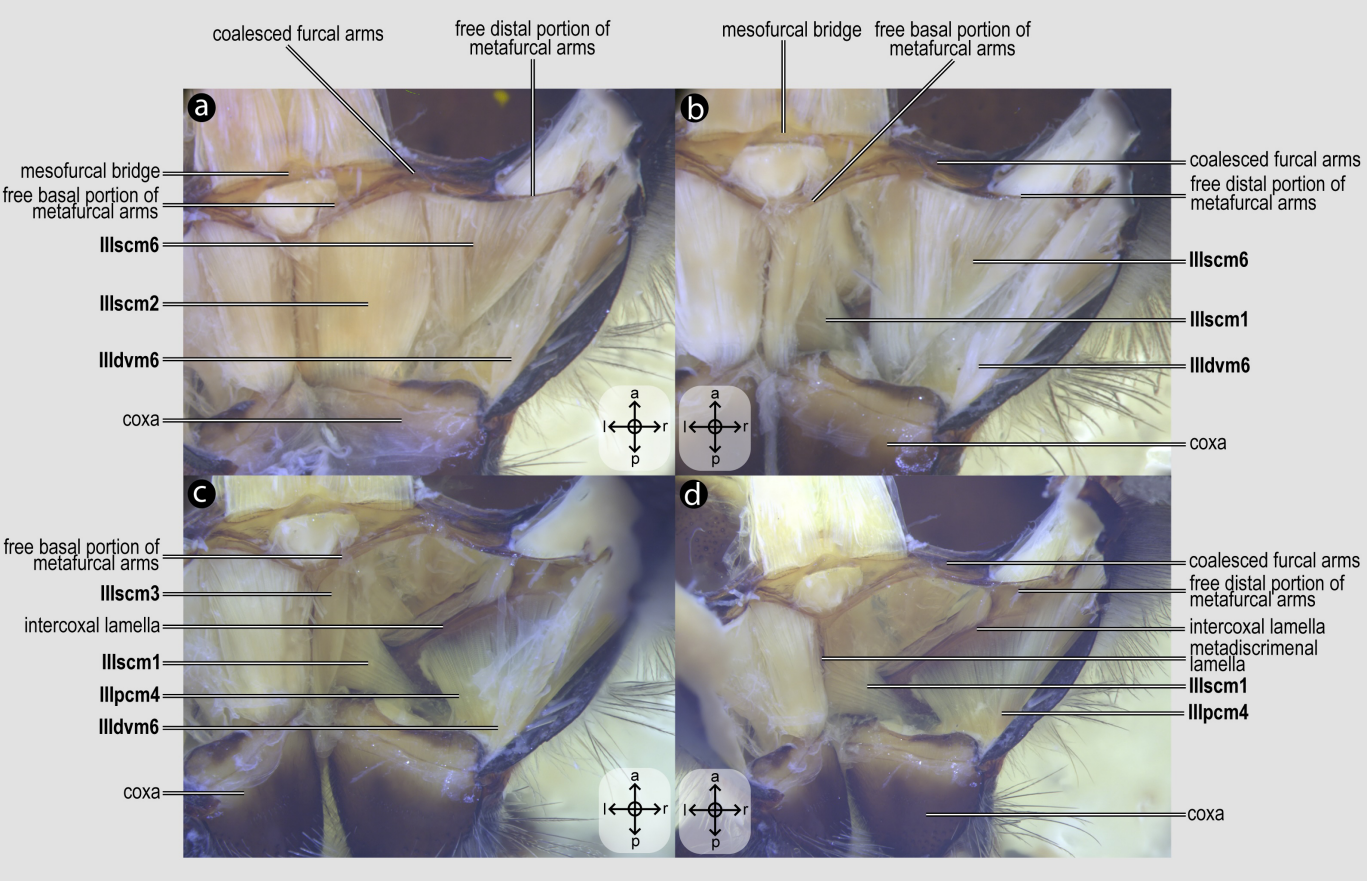
Metacoxal muscles. Metacoxal muscles of *Melipona quadrifasciata*: (A), (B), (C), (D) metacoxal muscles associated with the meso/metafurca—dorsal view of the metafurca. Muscle labels are displayed in bold and larger font to differentiate them from labels applied to sclerites and body regions; photomicrographs not to scale. The star depicted in (A) indicates structure orientation according to two axes: anterior–posterior and left–right.

**Figure 6:**
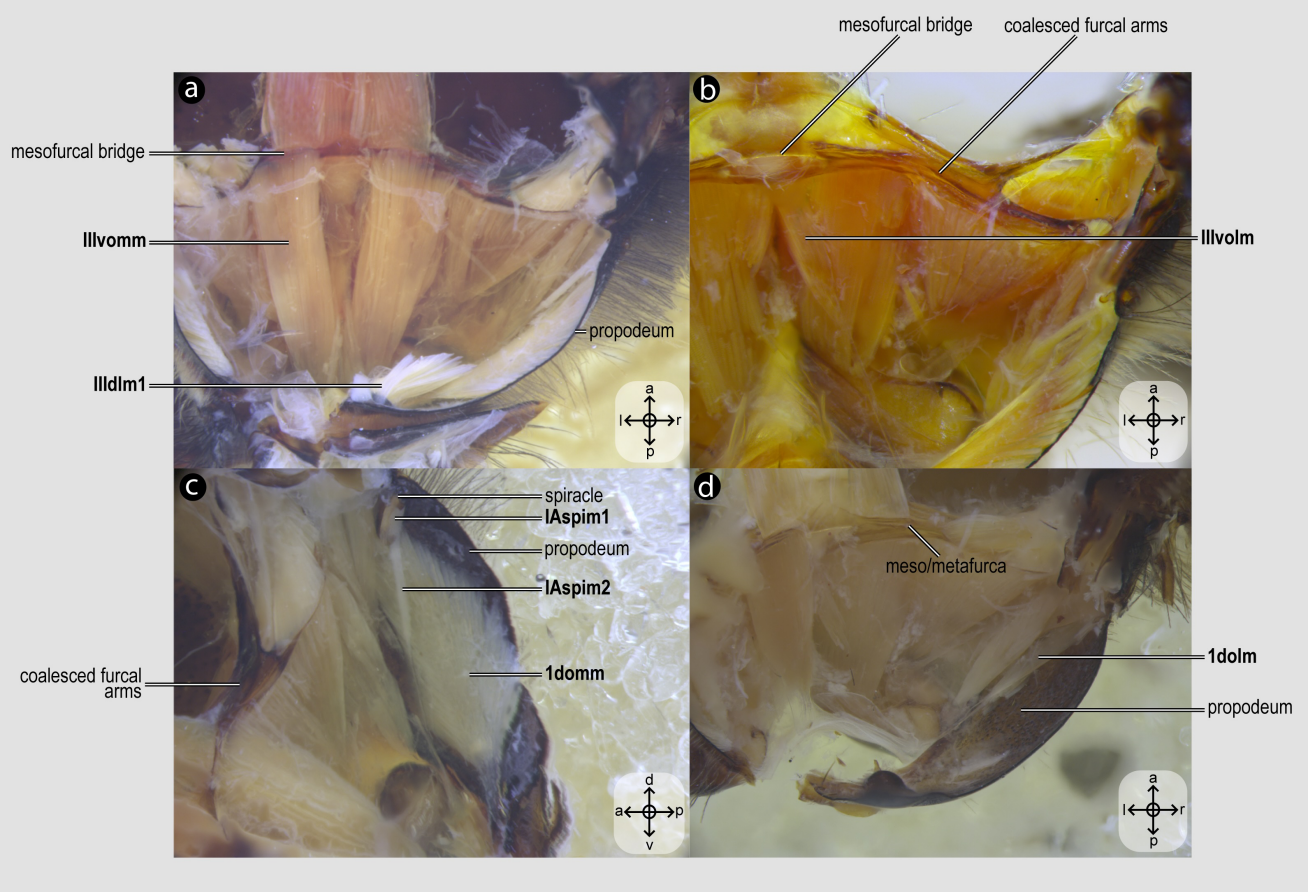
Propodeal muscles. Propodeal muscles of *Melipona quadrifasciata*: (A) propodeal muscles associated with the metafurca—dorsal view of metafurca; (B) propodeal muscles associated with metafurca—dorsolateral view of metafurca; (C) propodeal and spiracular muscles associated with the propodeum—medial view of the propodeum; (D) propodeal muscles—dorsal view of the metafurca. Muscle labels are displayed in bold and larger font to differentiate them from labels applied to sclerites and body regions; photomicrographs not to scale. Stars indicate structure orientation according to three axes: anterior–posterior, dorsal–ventral, and left–right.

Each dissection began with an incision along the margins of the mesoscutum, which was carefully removed to expose the indirect flight muscles (Fig. 2A). Dissections were performed in absolute ethanol within Petri dishes containing fine sand to stabilize the specimen, using microforceps and fine scalpels under a stereomicroscope to preserve delicate muscle attachments. Each muscle was meticulously dissected and photographed, before removal to progressively access deeper structures, documenting muscle attachments to skeletal elements and their spatial arrangements. This systematic documentation ensured a comprehensive record of the structural relationships and morphological details of the musculature. Next, an incision was made to remove the propectus (Fig. 3F), allowing access to the prothoracic musculature, including muscles associated with the forelegs and their articulation with the mesothorax and head. The study then proceeds posteriorly, examining the mesothoracic and metathoracic muscles (Figs. 4–6), focusing on the intrinsic and extrinsic muscle groups responsible for wing movement and structural support. Throughout this posterior dissection, the gradual removal of muscles continues, ensuring that each newly exposed structure is documented and analyzed *in situ* before proceeding further.

### Visualization and image acquisition

The musculature was observed in Petri dishes containing absolute ethanol filled with fine sand substrate to prevent the material from moving excessively. We used a Leica M205c stereomicroscope with transmitted and incident light. Images were taken with a Leica DFC450 camera using bright field illumination. The stack of images was obtained with Helicon Focus software (Helicon Soft Ltd., Kharkiv, Ukraine).

### Terminology

The terminology for the muscles and skeleton primarily follows Meira et al. (2024). The propectus is understood as the complex including the propleuron plus prosternum (per Snodgrass, 1942). The function of each muscle group mainly follows the interpretations of Snodgrass (1942), with additions by Mikó et al. (2007) and Vilhelmsen, Mikó & Krogmann (2010). Additional literature concerning the morphology of the mesosomal extrinsic musculature of several hymenopteran taxa was reviewed to contextualize the findings (Snodgrass, 1942; Vilhelmsen, 2000a; Vilhelmsen, 2000b; Vilhelmsen, Mikó & Krogmann, 2010; Mikó et al., 2007; Friedrich & Beutel, 2008; Willsch et al., 2020; Yoder et al., 2010; Aibekova et al., 2022; Lieberman et al., 2022), as summarized in the tab “*Terminology*” Table S1.

### Character coding and phylogenetic optimization

We constructed characters to assess the homology hypotheses implied by the similarities and variations found in the comparative morphological investigation of the mesosomal musculature. To that end, the character state transformations were mapped onto a pre-existing phylogenetic hypothesis using Fitch optimization (Fitch, 1971), as implemented in the software Winclada v.1.00.08 (Nixon, 2002). The ancestral reconstructions were analyzed under both unambiguous and ACCTRAN schemes, with the latter prioritizing early character transformations consistent with bee phylogeny, thus suggesting potential homologies (Agnarsson & Miller, 2008). For the character transformations, a summary tree including all 10 bee species of this study was prepared in Mesquite (Maddison & Maddison, 2007) by pruning the phylogenetic hypotheses of Almeida et al. (2023) for these 10 species, then complemented by the tree of Sann et al. (2018) to represent the three taxa of apoid wasps also investigated.

## Results

### Description of the skeletomusculature

**Figure 7:**
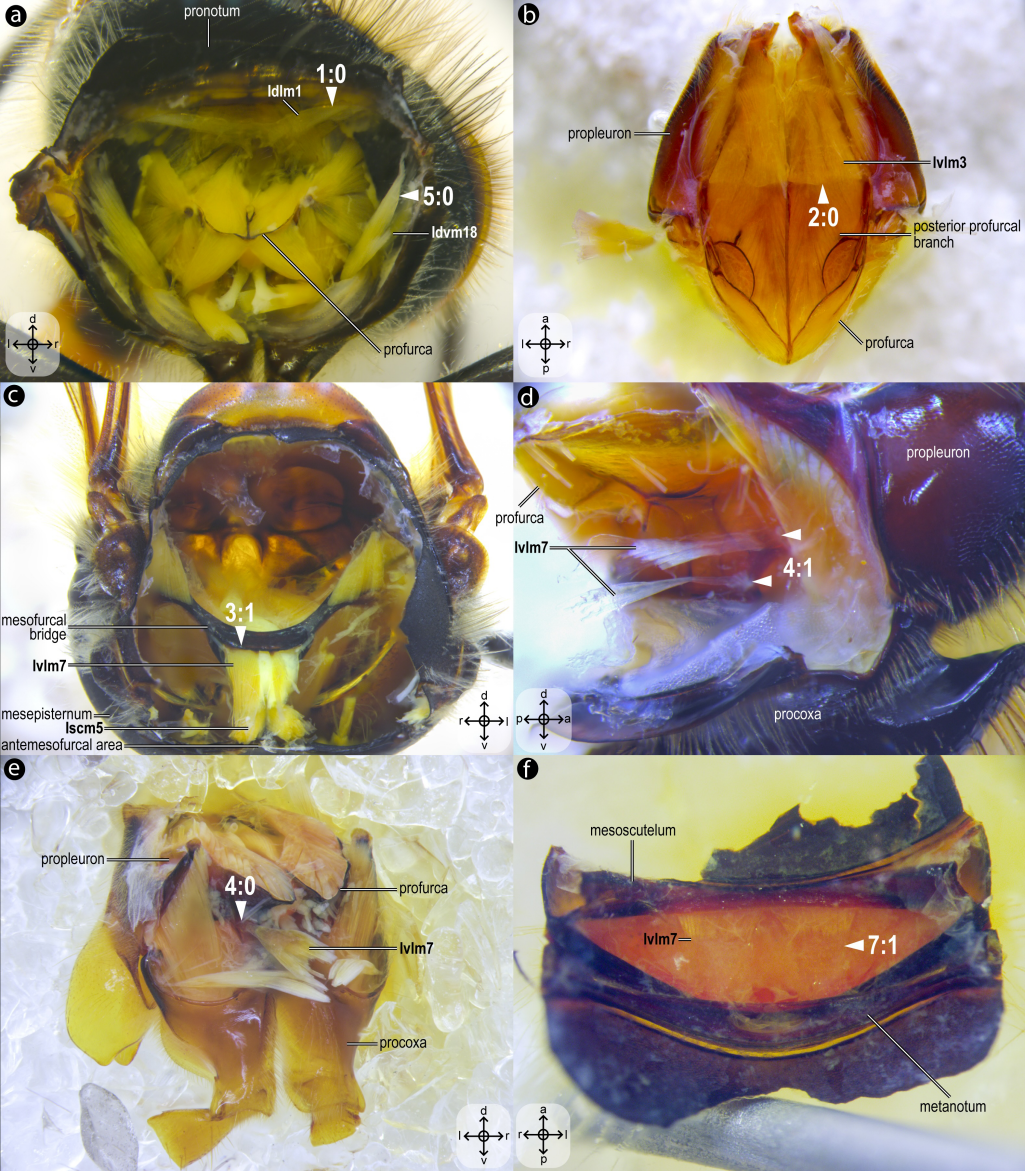
Variation of thoracic skeletomusculature: Part 1. Photomicrographs of dissections of female representatives of Apoidea: (A) *Apis mellifera*, posterior view of the propectus; (B) *Oxaea flavescens*, dorsal view of the propectus; (C) *Apis mellifera*, anterior view of the mesosoma; (D) *Lithurgus huberi*, lateral view of the profurca; (E) *Trypoxylon lactitarse*, posterolateral view of the propectus; (F) *Apis mellifera*, ventral view of mesoscutellum. Arrowheads indicate morphological conditions coded as character states. Stars indicate structure orientation according to three axes: anterior–posterior, dorsal–ventral, and left–right.

**Figure 8:**
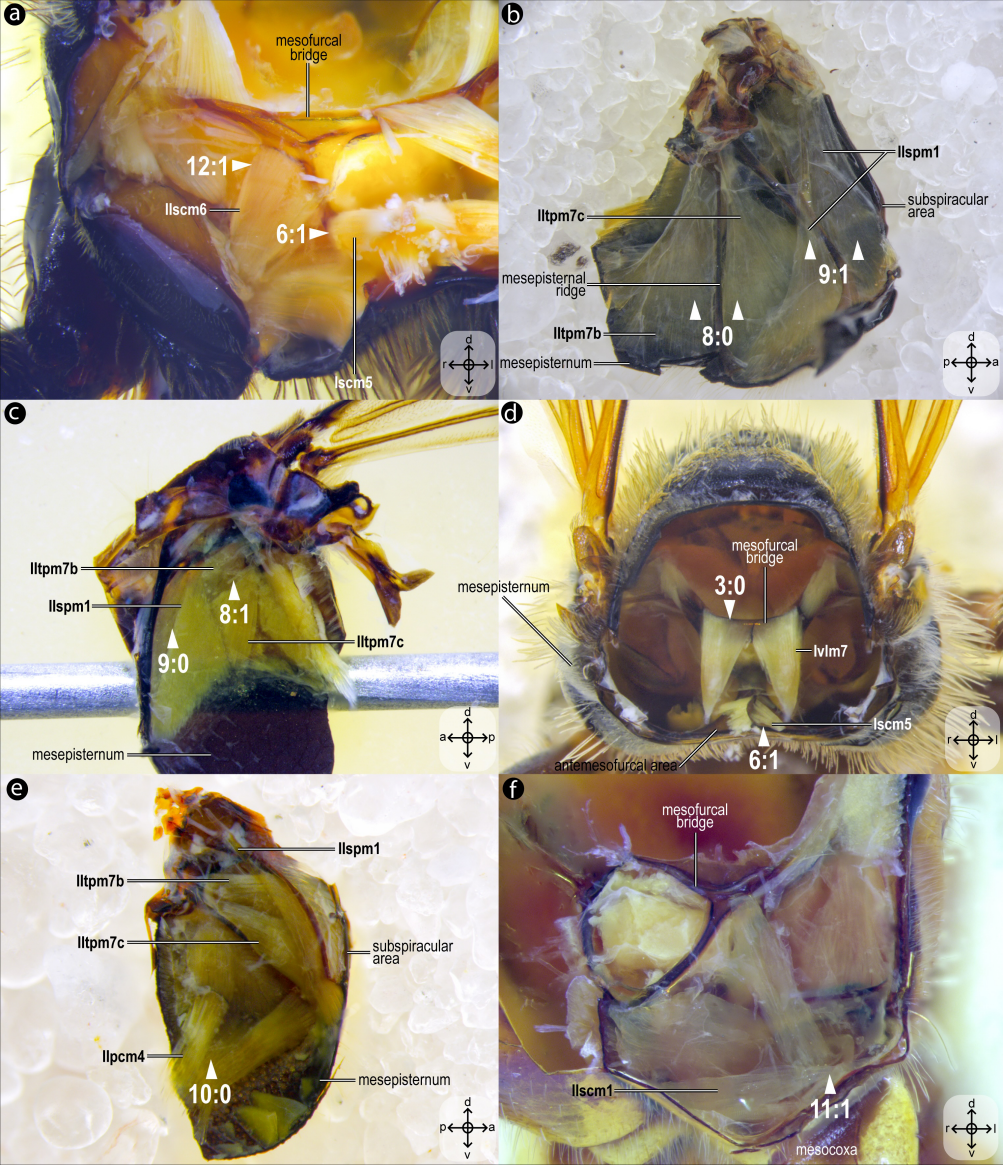
Variation of thoracic skeletomusculature: Part 2. Dissections of female representatives of Apoidea: (A) *Lithurgus huberi*, anterolateral view of the meso/metafurca; (B) *Trypoxylon lactitarse*, mesal view of the mesepisternum; (C) *Apis mellifera*, mesal view of the mesepisternum; (D), *Hesperapis carinata*, anterior view of the mesosoma; (E) *Psaenythia bergii*, mesal view of the mesepisternum; (F) *Trachypus boharti*, anterolateral view of meso/metafurca. Arrowheads indicate morphological conditions coded as character states. Stars indicate structure orientation according to three axes: anterior–posterior, dorsal–ventral, and left–right.

**Figure 9:**
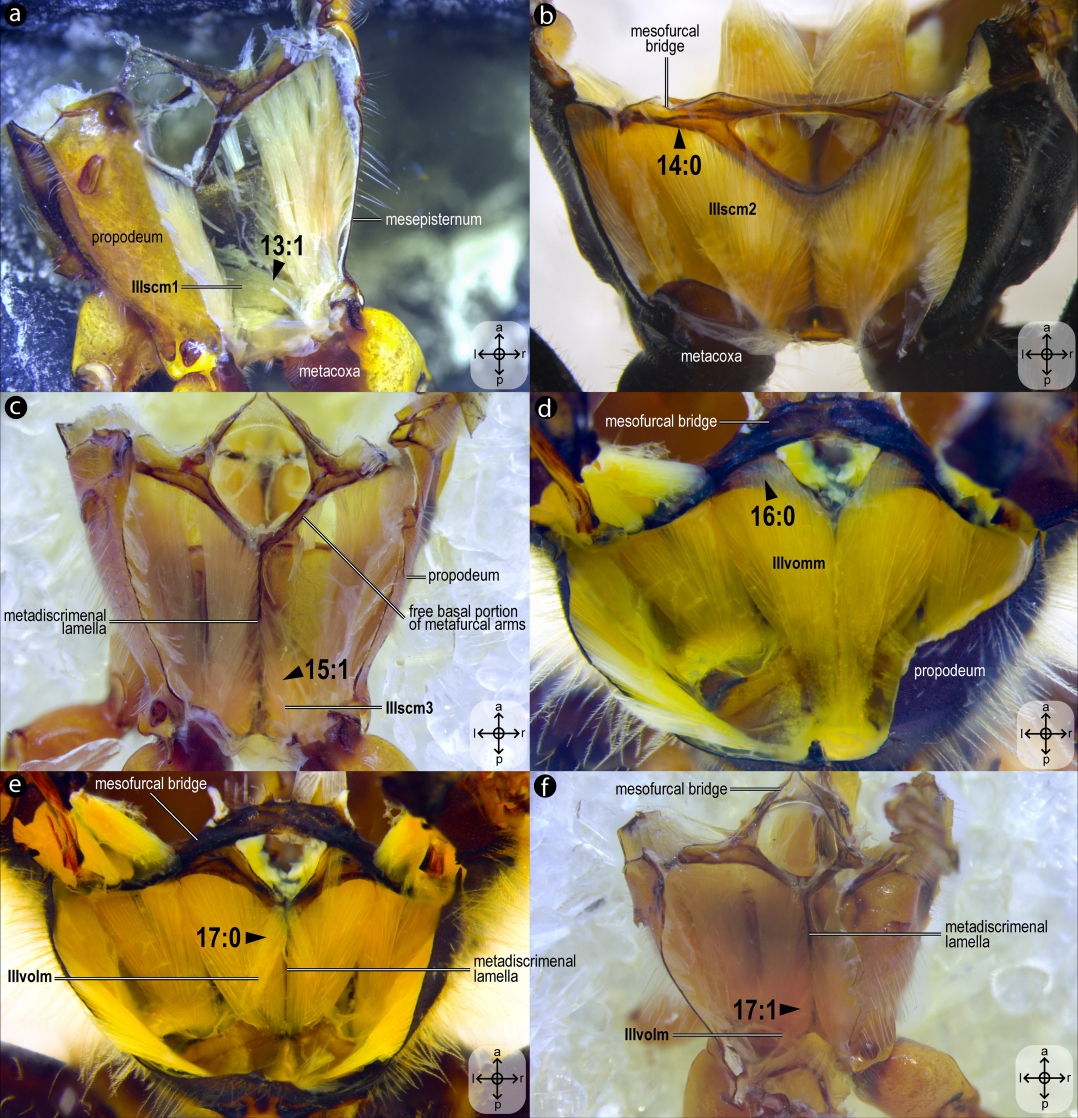
Variation of thoracic skeletomusculature: Part 3. Dissections of female representatives of Apoidea: (A) *Trachypus boharti*, dorsolateral view of the propodeum; (B) *Psaenythia bergii*, dorsal view of the propodeum; (C) *Trachypus boharti*, dorsal view of the propodeum; (D) *Apis mellifera* dorsal view of the propodeum; (E) *Apis mellifera*, dorsal view of the propodeum; (F) *Trachypus boharti*, dorsal view of the propodeum. Arrowheads indicate morphological conditions coded as character states. Stars indicate structure orientation according to two axes: anterior–posterior and left–right.

The variation of the mesosomal musculature of Apoidea is described in detail below based on ten species of bees and three apoid wasps. Figure 2–6 serve as an anatomical atlas of the main muscle groups and the dissections used to expose them; these are complemented by Figs. 7–9, which show the variations of some muscles among the species considered in this comparative analysis. The exemplars of these 13 taxa that were analyzed in this comparative research have the same number of muscle groups, although variations in size, relative position in relation to other muscles and sclerites, and their position of insertion and/or origin were observed for 16 of 58 studied muscles (intrinsic leg muscles were not studied). In instances where some degree of variation was documented, this is indicated by the “**VAR**” annotation at the muscle description; likewise, if the observed variation was used as the basis for character construction (see next section, below), the character number is indicated too. In contrast, no significant variation was documented for 42 muscle groups, and a concise summary is provided for those too; the lack of variation in such cases is indicated by the “**INVAR**” annotation in the muscle description. This variation, or lack thereof, is summarized in the *‘Variation’* tab of Table S1 and is detailed for each muscle below. The taxonomic distribution of this variation is shown by the character states coded for the species included in the study (see section ‘Character statements’ below and Table S2).

***Idlm1**, M. prophragma-occipitalis*(Figs. 3A–3B) [**VAR**: variation coded into character 1, below]: one of the five elevators of the head. It originates dorsomedially or dorsolaterally from the prophragma of bees, but always lateral to **Idlm2** (Figs. 3A, 3B) and inserts dorsolaterally on the postocciput. All three apoid wasps have Idlm1 located dorsolaterally on the prophragma, lateral to **Idlm2**, inserting on the dorsolateral areas of the postocciput. There is variation related to the origin point of this muscle (the insertion point does not vary in Apoidea). In *M*. *quadrifasciata*, *T*. *fiebrigi*, *S*. *quadripunctata*, and apoid wasps, the muscle origin is dorsomedially (Figs. 3A–3B) located on the prophragma. In contrast, the remaining bees have the muscle origin of **Idlm1** dorsolaterally (Fig. 7A) located on the prophragma. The morphology of Idlm1 observed in Meliponini suggests that the morphology of **Idlm1** might be a synapomorphy for the tribe.

***Idlm2**, M. pronoto-occipitalis*(Figs. 3A, 3B) [**INVAR**]: one of the five elevators of the head. The origin point is dorsomedially located on the posterior margin of the pronotum, and the insertion is dorsomedially located on the postocciput.

***Itpm1**, M. pleurocrista-occipitalis*(Figs. 3B, 3D) [**INVAR**]: one of the five elevators of the head. It originates from the propleuron (dorsal propleural margin) and inserts dorsolaterally on the postocciput through a shared tendon with the branched **Itpm2** (**Itpm2a** and **Itpm2b**, see below).

***Itpm2**, M. propleuro-occipitalis*(Fig. 3D) [**INVAR**]: one of the five elevators of the head. Branched muscle (**Itpm2a**, M. propleuro-occipitalis dorsal and **Itpm2b**, M. propleuro-occipitalis ventral); both branches originating from the ventral propleura area and inserting dorsolaterally to the foramen magnum on the postocciput through a shared tendon with **Itpm1**.

***Idvm9**, M. profurca-occipitalis*(Figs. 3B, 3C) [**INVAR**]: one of the five elevators of the head. Large muscle broadly originating from the profurca (anterodorsal and posterodorsal profurcal lamellae on the posterior profurcal branch), inserting dorsolaterally to the foramen magnum (close to the insertion of **Itpm1** and **Itpm2**) on the postocciput.

***Ivlm3**, M. profurca-tentorialis*(Fig. 3C) [**VAR**: variation coded into character 2, below]: the only depressor of the head. Large muscle whose origin can be located either on the dorsal (Fig. 3C) or the ventral (Fig. 7B) surface of the posterior profurcal branch, and the insertion is located ventrolaterally on the postocciput. Two bee species (*O*. *divaricatus* [Halictidae] and *H*. *carinata* [Melittidae]) have this ventral origin, while the remaining bees and apoid wasps have a dorsal origin.

***Idlm5**, M. pronoto-phragmalis anterior*(Fig. 3A) [**INVAR**]: a depressor of the pronotum. Broad and short muscle originating laterally from the inner surface of the pronotum and inserting laterally on the prophragma of the mesoscutum.

***Idvm5**, M. pronoto-cervicalis anterior*(Fig. 3A) [**INVAR**]: the elevator of the propleuron. Branched muscle (**Idvm5a**, M. pronoto-cervicalis anterior primus and **Idvm5b**, M. pronoto-cervicalis anterior secundus) with two well-separated origins on the pronotum: one dorsolateral (**Idvm5a**) and one dorsomedial (**Idvm5b**). The insertion of both branches is located on the cervical apodeme of the propleuron.

***Itpm3**, M. pronoto-pleuralis anterior*(Fig. 3A) [**INVAR**]: protractor of the propectus. Broad pronotal muscle, with its origin dorsomedially on the pronotum, close to the origin of **Idvm5b**, and its insertion on the anterior lamella of the dorsal propleural margin.

***Itpm4**, M. pronoto-apodemalis anterior*(Fig. 3A) [**INVAR**]: protractor of the propectus. Muscle originating anterolaterally from the pronotum and inserting distally on the propleural arm of the propleuron.

***Itpm5**, M. pronoto-apodemalis posterior*(Fig. 3A) [**INVAR**]: protractor of the propectus. Muscle originating posterolaterally from the pronotum and inserting distally on the propleural arm of the propleuron.

***Ivlm1**, M. profurca-cervicalis*(Fig. 3C) [**INVAR**]: the adductor of the propleuron. Muscle originating from the anteromedian profurcal process of the prosternum and inserting posteriorly on the cervical apodeme of the propleuron.

***Ivlm7**, M. profurca-mesofurcalis*(Figs. 2D, 4C) [**VAR**: variation coded into characters 3 and 4, below]: the retractor of the propectus. Intersegmental muscle originating broad (Figs. 2D, 4C, 8D) or narrowly (Fig. 7C) from the mesofurcal bridge of the meso-metafurca and inserting in one (Fig. 7E) or two scars (Fig. 7D) on the posterior surface of the profurca at the prosternum. Only *A*. *mellifera* and *T*. *fiebrigi* (Apidae) have a narrow origin on the mesofurcal bridge. Three species, *H*. *carinata* (Melittidae), *O*. *divaricatus* (Halictidae), and *L*. *huberi* (Megachilidae), have two insertion points on the profurca.

***Ipcm2**, M. procoxa cervicalis transversalis*(Figs. 3C, 3E) [**INVAR**]: rotator of the procoxa. Muscle originating from the anteromedian bar of the cervical apodeme of one side and inserting on the anterolateral margin of the procoxal base of the opposite side.

***Ipcm4**, M. propleuro-coxalis superior*(Fig. 3E) [**INVAR**]: lateral promotor of the procoxa. Muscle originating from the anterior process of the dorsal profurcal lamella and from the ventral surface of the dorsal propleural margin. The insertion is located on the anterolateral margin of the procoxal base.

***Iscm1**, M. profurca-coxalis anterior*(Fig. 3E) [**INVAR**]: medial promotor of the procoxa. Muscle originating from the prodiscrimenal lamella of the prosternum and inserting anteromedially on the procoxal base.

***Idvm18**, M. pronoto-coxalis lateralis*(Fig. 3A) [**VAR**: variation coded into character 5, below]: lateral remotor of the procoxa. Long muscle that originates laterally (Fig. 7A) or dorsolaterally (Fig. 3A) from the pronotum, close to the pronotal lobe, and inserts posterolaterally on the procoxal base. The morphology of **Idvm18** observed in *A*. *mellifera*, in which this muscle originates laterally on the pronotum, is interpreted as an autapomorphy of the species.

***Iscm3**, M. profurca-coxalis medialis*(Figs. 3A, 3F) [**INVAR**]: medial remotor of the procoxa. Thin muscle that originates from the sheet of the propleural arm and inserts posteromedially on the procoxal base.

***Iscm4**, M. profurca-coxalis lateralis*(Figs. 3A, 3F) [**INVAR**]: lateral remotor of the procoxa. Broad muscle that originates from the posterodorsal profurcal lamella of the profurcal arm and inserts posterolaterally on the procoxal base.

***Iscm5**, M. prospina-coxalis*(Figs. 3A, 4D) [**VAR**: variation coded into character 6, below]: retractor of the propectus. Muscle with origin varying from restricted to the horizontal plate of the meso/metafurca (Figs. 4D, 7C, 8D) to broader and also reaching the free basal portion of mesofurcal arms (Fig. 8A). The insertion is located posteriorly on the procoxal base. The morphology of **Iscm5** observed in *L*. *huberi*, in which the origin reaches the free basal portion of the mesofurcal arms, is interpreted as an autapomorphy of this species.

***Iscm6**, M. profurca-trochanteralis*(Figs. 3A, 3F) [**INVAR**]: depressor of the protrochanter. Broad muscle originating from the sheet of the propleural arm and inserting on the depressor tendon of the protrochanter.

***IIIdlm3**, M. metascutello-scutellaris*(Fig. 2B) [**VAR**: variation coded into character 7, below]: retractor of the mesoscutellum. Muscle originating medially (Fig. 7E) or laterally (Fig. 2B) from the scutoscutellar ridge of the mesoscutellum and the insertion located medially or laterally on the anterior margin of the internal metanotal ridge. The morphology of **IIIdlm3** observed in *A*. *mellifera*, in which both origin and insertion of this muscle are located laterally, is interpreted as an autapomorphy of the species.

***IIdlm1**, M. prophragma-mesophragmalis*(Fig. 2A) [**INVAR**]: indirect depressor of the wing. Large dorsal longitudinal indirect flight muscle with the origin medially located on the ventral side of the mesoscutum and on the posterior face of the prophragma, and the insertion is located broadly on the anterior face of the mesophragma.

***IIdvm1**, M. mesonoto-sternalis*(Fig. 2A) [**INVAR**]: indirect elevator of the wing. Mesonotal muscle that arises laterally from the ventral side of the mesoscutum and from the posterior margin of the prophragma and inserts broadly on the mesepisternum.

***IIpspim1**, M. mesanepisterno-spiracularis*(Fig. 2C) [**INVAR**]: occlusor of the anterior thoracic spiracle. Muscle of the mesothorax, originating from the anterior margin of the subspiracular area and inserting on the spiracular membrane at the spiracular aperture.

***IItpm5**, M. mesonoto-pleuralis medialis*(Fig. 4A) [**INVAR**]: depressor of the mesoscutellum. Muscle originating from the lateral areas of the mesepisternal region and from the pleural apophysis; its insertion is located on the lateral margin of the mesoscutellum.

***IItpm7**, M. mesanepisterno-axillaris*(Fig. 4A): flexors of the forewing (***IItpm7a***, *M. mesanepisterno-axillaris ventral*, ***IItpm7b**, M. mesanepisterno-axillaris medial*and ***IItpm7c**, M. mesanepisterno-axillaris dorsal*) [**VAR**: variation coded into character 8, below]: **IItpm7a** originates from the subalar apophysis cavity, while **IItpm7b** and **IItpm7c** originate laterally from the mesepisternum; all three branches insert, by a shared tendon, on the third mesoaxillary sclerite. In bees, the absence of separation between **IItpm7b** and **IItpm7c** by the mesepisternal ridge (Figs. 4A, 8C) is likely a synapomorphy, as among apoid wasps most species, except the Bembicidae species, have this separation (Fig. 8B).

***IIspm1**, M. mesopleura-sternalis*(Fig. 4A) [**VAR**: variation coded into character 9, below]: depressor of the mesobasalar sclerite. Muscle that originates from the anterior margin of the mesepisternum (Fig. 8C) and/or the subspiracular area (Figs. 4A, 8B) and inserts on the mesobasalar sclerite. The morphology of **IIspm1** observed in *A*. *mellifera*, in which this muscle origin is restricted to the anterior region of the mesepisternum, is interpreted as an autapomorphy of the species.

***IIspm2**, M. mesofurca-pleuralis*(Figs. 4C–4D) [**INVAR**]: furco-pleural muscle with uncertain function. Muscle originates from the posterolateral area of the mesepisternum and inserts on the tip of the free distal portion of the mesofurcal arm.

***IIpcm4**, M. propleuro-coxalis posterior*(Fig. 4D) [**VAR**: variation coded into character 10, below]: lateral promotor of the mesocoxa. Muscle origin branched (Fig. 8E) or not (Fig. 4D), located laterally on the mesepisternum; insertion located laterally on the mesocoxal base. The variation of origin point of **IIpcm4** suggests multiple independent changes in Apoidea, further suggesting a complex evolution of this muscle. This variation was already described by Wille (1956), although not coded as character and character states.

***IIscm1**, M. mesofurca-coxalis anterior*(Fig. 4C) [**VAR**: variation coded into character 11, below]: medial promotor of the mesocoxa. Muscle that originates posteroventrally from the mesodiscrimenal lamella and inserts antero- (Fig. 4C) or mediolaterally (Fig. 8F) on the mesocoxal base. In bees, the muscle consistently inserts anterolaterally on the mesocoxa, a feature also observed in the bembicid species but not in the other two apoid wasps studied.

***IIdvm6**, M. mesocoxa-subalaris*(Figs. 4C–4D) [**INVAR**]: lateral remotor of the mesocoxa. Slender muscle that originates posterolaterally from the mesocoxal base and inserts on the mesosubalar sclerite.

***IIscm2**, M. mesofurca-coxalis posterior*(Fig. 4C) [**INVAR**]: medial remotor of the mesocoxa. Muscle that originates anteroventrally from the mesodiscrimenal lamella of the mesofurca and inserts posteromedially on the base of the mesocoxa.

***IIscm6**, M. mesofurca-trochanteralis*(Fig. 4C) [**VAR**: variation coded into character 12, below]: thoracic depressor of the mesotrochanter. Broad muscle with origin restricted to the coalesced furcal arms (Fig. 4C) or also reaching free basal portion of mesofurcal arms (Fig. 8A). Insertion located on the depressor tendon of the trochanter. The morphology of IIscm6 observed in *M*. *quadrifasciata*, in which this muscle is restricted to the coalesced furcal arms, is interpreted as an autapomorphy of that species.

***IIIdlm1**, M. mesophragma-metaphragmalis*(Fig. 6A) [**INVAR**]: retractor of the mesophragma. Longitudinal muscle of the mesophragma that originates from the propodeal ridge and inserts on the posterior surface of the mesophragma.

***IIItpm5**, M. metanoto-pleuralis medialis*(Fig. 4A) [**INVAR**]: one of the hindwing elevators. Muscle that originates from the free distal portion of the metafurcal arm and dorsal metafurcal lamella and inserts on the dorsolateral metanotal area.

***IIItpm6**, M. metanoto-pleuralis posterior*(Fig. 4A) [**INVAR**]: one of the hindwing elevators. Muscle that originates from the dorsal metafurcal lamella and inserts on the tip of the dorsolateral metanotal area.

***IIIdvm1**, M. metanoto-sternalis*(Fig. 4A) [**INVAR**]: one of the hindwing elevators. Tergosternal muscle of the metathorax that originates from the dorsolateral metanotal area and inserts on the filamentous process of the free distal portion of the metafurcal arm.

***IIItpm7**, M. metanepisterno-axillaris*(Figs. 4A, 4B) [**INVAR**]: flexor of the hindwing. Broad muscle with its origin located on the anterior inflection of the metepisternum and the insertion located on the third metaaxillary sclerite.

***IIIspm1**, M. metapleura-sternalis*(Fig. 4B) [**INVAR**]: depressor of the metabasalar sclerite. Muscle arising from the ventral region of the metapectus and inserting on the metabasalar sclerite.

***IIItpm11**, M. metapleura-subalaris*(Fig. 4B) [**INVAR**]: depressor of the metasubalar sclerite. Muscle arising from the anterior inflection of the metapectus and inserting on the metasubalar sclerite.

***IIIpcm4**, M. metanepisterno-coxalis posterior*(Figs. 5C–5D) [**INVAR**]: lateral promotor of the metacoxa. Muscle that originates broadly from the intercoxal lamella and the metapleural ridge and inserts anterolaterally on base of the metacoxa.

***IIIdvm6**, M. metacoxa-subalaris*(Figs. 5A–5C) [**INVAR**]: lateral remotor of the metacoxa. Muscle of the metathorax originating posterolaterally from the metacoxal base and inserting on the metasubalar sclerite.

***IIIscm1**, M. metafurca-coxalis anterior*(Figs. 5B–5D) [**VAR**: variation coded into character 13, below]: medial promotor of the metacoxa. Broad muscle that originates from the intercoxal lamella, reaching (Figs. 5B–5D), or not (Fig. 9A), the free basal portion of the metafurcal arms and inserting anteriorly on the metacoxal base. The morphology of IIIscm1 reaching the free basal portion of the metafurcal arms is observed in bees and *Steniolia duplicata* (Bembicidae).

***IIIscm2**, M. metafurca-coxalis posterior*(Fig. 5A) [**VAR**: variation coded into character 14, below]: posterior remotor of the metacoxa. Muscle that originates from the free basal portion of the metafurcal arm, sometimes extending to the coalesced furcal arms (Fig. 9B) or not (Fig. 5A), and inserts posteriorly on the metacoxal base. The morphology of **IIIscm2** observed in all members of Andrenidae, Colletidae, and Halictidae, in which this muscle origin extends to the coalesced furcal arms, is likely a synapomorphy of the clade formed by these three bee families.

***IIIscm3**, M. metafurca-coxalis medialis*(Fig. 5C) [**VAR**: variation coded into character 15, below]: medial remotor of the metacoxa. Muscle that originates from the metadiscrimenal lamella, sometimes extending to the free basal portion of the metafurcal arms (Fig. 5C) or not (Fig. 9C), and inserts posteromedially on the metacoxal base. The morphology of **IIIscm3** reaching the free basal portion of the metafurcal arms observed in all bees and *T*. *lactitarse* (Crabronidae).

***IIIscm6**, M. metafurca-trochanteralis*(Figs. 5A–5B) [**INVAR**]: thoracic depressor of the metatrochanter. Muscle that originates from the posterior surface of the mesofurcal bridge and coalesced furcal arms and inserts on the depressor tendon of the metatrochanter.

***IIIvomm**, M. metafurca-abdominosternalis medialis*(Fig. 6A) [**VAR**: variation coded into character 16, below]: medial depressor of the abdomen. Muscle that originates from the free basal portion of the metafurcal arm, sometimes extending to the coalesced furcal arms (Fig. 6A) or not (Fig. 9D), inserting medially on the first metasomal segment. While its insertion remains consistent across species, its origin varies in *T*. *fiebrigi*, *S*. *quadripunctata*, *A*. *mellifera*, and *H*. *carinata*, extending to the coalesced furcal arms, whereas in the remaining analyzed species, it is restricted to the free basal portion of the metafurcal arm. This variation was already described by Wille (1956), although not coded as character and character states.

***IIIvolm**, M. metafurca-abdominosternalis lateralis*(Fig. 6B) [**VAR**: variation coded into character 17, below]: ventral lateromotor of the abdomen. Muscle that originates from the metadiscrimenal lamella and inserts on the sternum of the first metasomal segment. While its insertion remains consistent across species, its origin varies: in Apidae and Oxaeinae, it is positioned anteriorly (Figs. 6B, 9E) on the metadiscrimenal lamella, whereas in all remaining species, it is located posteriorly (Fig. 9F).

***1domm**, M. tergo-tergalis orthomedialis*(Fig. 6C) [**INVAR**]: medial elevator of the abdomen. Muscle that originates broadly from the anterior wall of the propodeum and inserts medially on the constricted margin of the first metasomal tergum.

***1dolm**, M. tergo-tergalis ortholateralis*(Fig. 6D) [**INVAR**]: dorsal lateromotor of the abdomen. Muscle that originates from the lateral wall of the propodeum and inserts laterally on the anterior margin of the first metasomal tergum.

***IAspim1**, M. spiracularis I superior*(Fig. 6C) [**INVAR**]: occlusor of the propodeal spiracle. Muscle of the propodeal spiracle, originating from the sclerotized area above the propodeal spiracle and inserting on the sclerotized area below the propodeal spiracle.

***IAspim2**, M. spiracularis I posterior*(Fig. 6C) [**INVAR**]: dilator of the propodeal spiracle. Muscle of the propodeal spiracle originating from the small metapleural coxal process and inserting on the sclerotized area below the propodeal spiracle.

### Character statements

Below are described the 17 morphological characters and their respective states derived from the extrinsic mesosomal musculature of bees, which were scored for the 13 species of Apoidea (ten bee and three wasp species) (Table S2). These characters summarize the overall morphological variation described in the previous section and are interpreted in a phylogenetic context being optimized onto a phylogenetic hypothesis for bees.

01. Origin of **Idlm1**: (0) dorsolaterally at the prophragma (Fig. 7A); (1) dorsomedially at the prophragma (Figs. 3A–3B).

02. Origin of **Ivlm3**: (0) ventrally at the posterior profurcal branch (Fig. 7B); (1) dorsally at the posterior profurcal branch (Fig. 3C).

03. Origin of **Ivlm7**: (0) broadly at the mesofurcal bridge (Figs. 2D, 4C, 8D); (1) narrowly, restricted to the ventral margin of the mesofurcal bridge (Fig. 7C).

04. Insertion of **Ivlm7**: (0) single, at the posterior face of the profurcal arm (Fig. 7E); (1) two, at the posterior face of the profurcal arm (Fig. 7D).

05. Origin of **Idvm18**: (0) laterally at the pronotum (Fig. 7A); (1) dorsolaterally at the pronotum (Fig. 3A).

06. Origin of **Iscm5**: (0) restricted to the horizontal plate of the meso/metafurca (Figs. 4D, 7C, 8D); (1) reaching the free basal portion of the mesofurcal arms (Fig. 8A).

07. Insertion of **IIIdlm3**: (0) medially at the anterior margin of the internal metanotal ridge (Fig. 7F); (1) laterally at the anterior margin of the internal metanotal ridge (Fig. 2B).

08. Origin of **IItpm7b** and **IItpm7c**: (0) separated by the mesepisternal ridge (Fig. 8B); (1) not separated by the mesepisternal ridge (Figs. 4A, 8C).

09. Origin of **IIspm1**: (0) at the anterior margin of the mesopectus (Fig. 8C); (1) at the anterior margin of the mesopectus and at the subalar area (Figs. 4A, 8B).

10. Origin of **IIpcm4**: (0) branched (Fig. 8E); (1) not branched (Fig. 4D).

11. Insertion of **IIscm1**: (0) anterolateral (Fig. 4C); (1) mediolateral (Fig. 8F).

12. Origin of **IIscm6**: (0) restricted to the coalesced furcal arms (Fig. 4C); (1) reaching the free basal portion of the mesofurcal arms (Fig. 8A).

13. Origin of **IIIscm1**: (0) reaching the free basal portion of metafurcal arms (Figs. 5B–5D); (1) restricted to the metadiscrimenal lamella (Fig. 9A).

14. Origin of **IIIscm2**: (0) reaching the coalesced furcal arms (Fig. 9B); (1) restricted to the free basal portion of the metafurcal arms (Fig. 5A).

15. Origin of **IIIscm3**: (0) reaching the free basal portion of metafurcal arms (Fig. 5C); (1) restricted to the metadiscrimenal lamella (Fig. 9C).

16. Origin of **IIIvomm**: (0) broadly at the free distal portion of metafurcal arm (Fig. 9D); (1) at the free distal portion of metafurcal arms and coalesced furcal arms (Fig. 6A).

17. Origin of **IIIvolm**: (0) anteriorly at the metadiscrimenal lamella (Figs. 6B, 9E); (1) posteriorly at the metadiscrimenal lamella (Fig. 9F).

## Discussion

The mesosoma, a critical region in bees, exhibits remarkable structural conservation across this diverse insect group, as first highlighted by Wille (1956). This seminal work explored the evolutionary trends of ten key thoracic muscles, providing insights into their morphological consistency and variation. These muscles include the lateral promotor of the middle coxa (herein **IIpcm4**, M. propleuro-coxalis posterior; coded as character 10), the pleurotergal muscle of the mesothorax (herein **IItpm5**, M. mesonoto-pleuralis medialis; showing no significant variation in this study), the median depressor of the abdomen (herein **IIIvomm**, M. metafurca-abdominosternalis medialis; coded as character 16), the lateral promotor of the hind coxa (herein **IIIpcm4**, M. metanepisterno-coxalis posterior; lacking notable variation here) and the mesal remotor of the hind coxa (herein **IIIscm2**, M. metafurca-coxalis posterior, and **IIIscm3**, M. metafurca-coxalis medialis; where variation aligns with broader patterns of Hymenoptera, following Meira et al., 2024). This study builds on Wille’s (1956) findings, revealing that only 16 of 58 mesosomal muscle groups exhibit substantial variation across 10 bee and three apoid wasp taxa, reflecting a balance between phylogenetic conservatism and functional adaptation.

The limited variation, primarily in muscle origin points, underscores the mesosoma’s evolutionary stability, with insertion points remaining highly conserved. This pattern suggests that origin points are less functionally constrained, allowing evolutionary flexibility without compromising muscle function, as the insertion point primarily dictates movement mechanics (Snodgrass, 1935). One notable pattern of variation is observed in the muscle **Idlm1**. In bees, the muscle origin is either dorsomedially or dorsolaterally positioned on the prophragma, while apoid wasps consistently exhibit a dorsolateral origin. Among the examined stingless bee species, *M. quadrifasciata*, *T. fiebrigi*, and *S. quadripunctata* (Apidae: Meliponini), the dorsomedial origin appears to be a shared derived feature, suggesting a synapomorphy (char: 1, state: 1; Figs. 3A–3B, 10) for the tribe. Conversely, other bee taxa have a lateral origin (char: 1, state: 0; Figs. 7A, 10), indicating independent evolutionary shifts in **Idlm1**. Aibekova et al. (2022) noted that variations in thoracic muscle origins are likely linked to specific functional demands, such as movements of the head and the mesosomal appendages, further supporting the idea that the observed differences in **Idlm1** may reflect both phylogenetic history and biomechanical adaptations. These evolutionary morphological changes also support the findings of Vilhelmsen, Mikó & Krogmann (2010), when noting that mesosomal musculature can indicate both evolutionary history and functional adaptations.

**Figure 10:**
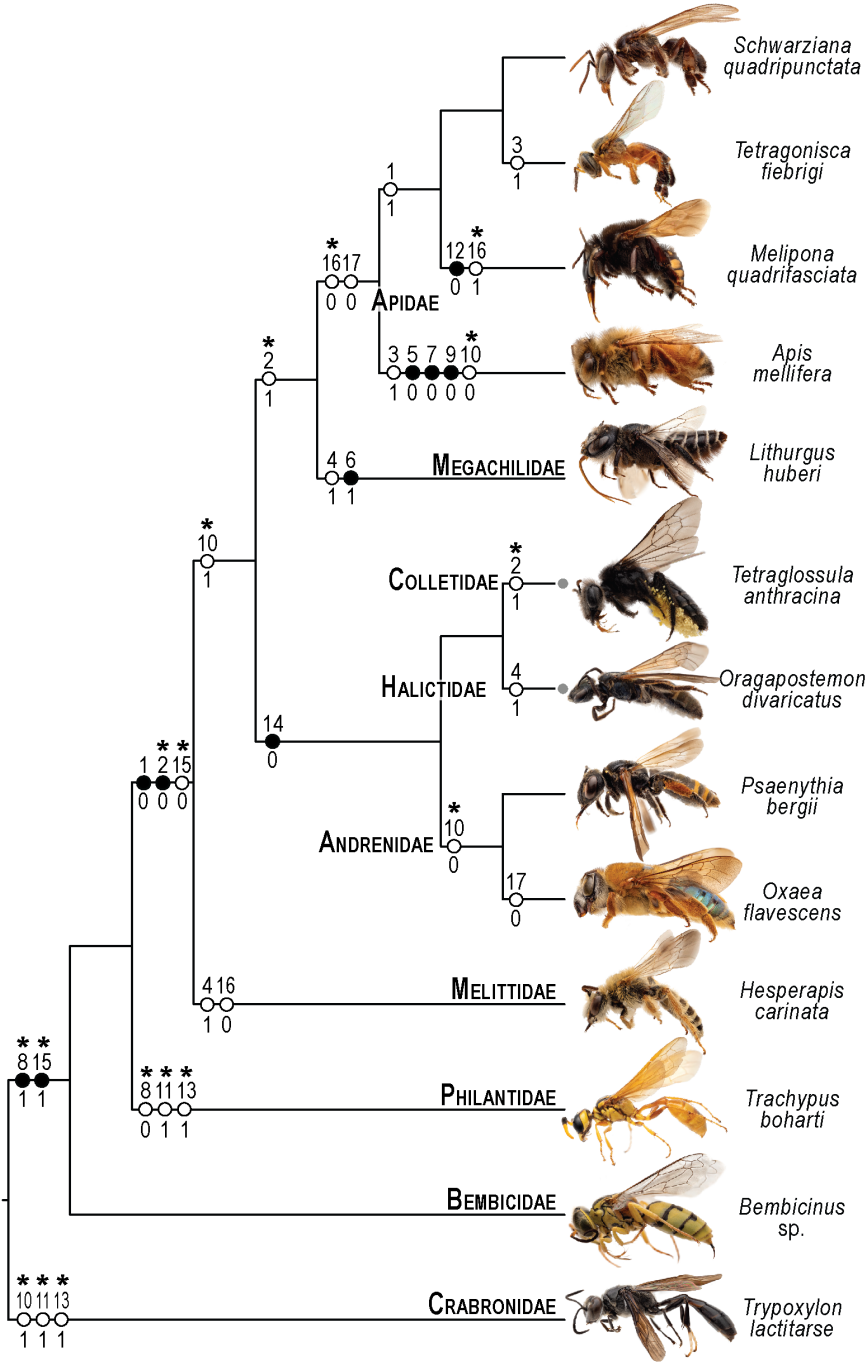
Phylogenetic interpretation of variation in the skeletomusculature of the mesosoma. Phylogenetic relationships among the 13 species of Apoidea investigated in this research follow Almeida et al. (2023) for relationships among bee taxa and Sann et al. (2018) for the three apoid wasp lineages. Evolutionary changes of 13 characters encoding variation in the skeletomusculature of the mesosoma were phylogenetically optimized onto the tree. Filled circles indicate unique transformations, while empty circles signify non-unique transformations; numbers above the circles denote character numbers, and those below indicate their apomorphic character states; ambiguous changes only recovered with accelerated transformation (ACCTRAN) are indicated by stars above their character numbers. Photographs on the left are not to scale and serve to illustrate the general appearance of each taxon (see Fig. 1 for more details).

Similarly, the variation in the muscle group **Ivlm3** suggests significant phylogenetic implications. While most bees exhibit a dorsal origin (char: 2, state: 1; Figs. 3C, 10), both andrenid species, as well as *O. divaricatus* [Halictidae] and *H. carinata* [Melittidae] retain a ventral origin (char: 2, state: 0; Figs. 7B, 10). This distribution suggests that the dorsal attachment may represent an ancestral condition preserved in specific lineages, whereas the ventral origin likely evolved independently in other groups. The ventral origin likely represents a derived condition, as interpreted by Vilhelmsen (2000b), potentially facilitating stronger head depression movements, such as those associated with certain foraging or ground-nesting behaviors. The dorsal origin, conversely, may enhance head flexibility as well as biomechanical demands for varied head positioning (Dudley, 2000).

Based on our results, apoid wasps and bees differ in the morphology of the **Ivlm7**. The extent of variation in Apoidea remains unexplored, but we sampled representatives of three taxa that represent at least a fraction of the diversity of apoid wasps. While most taxa exhibit a broad attachment to the mesofurcal bridge (char: 3, state: 0; Figs. 2D, 4C, 8D, 10), the species *A*. *mellifera* and *T*. *fiebrigi* display a restricted ventral attachment (char: 3, state: 1; Figs. 7C, 10). The restricted ventral attachment could be linked to an enhancement of the thoracic rigidity, supporting the high-frequency wing beats (Dickinson, 2006). Additionally, **Ivlm7** can have one (char: 4, state: 0; Figs. 7E, 10) or two (char: 4, state: 1; Figs. 7D, 10) insertions in bees, suggesting biomechanical adaptations for enhanced propectus retraction, potentially aiding in precise leg movements during nesting or pollen collection (Michener, 2007).

The metathoracic muscle **IIIscm2** (M. metafurca-coxalis posterior) exhibits a phylogenetically informative pattern, with an extended origin to the coalesced furcal arms in Andrenidae, Colletidae, and Halictidae (char: 14, state: 0; Figs. 9B, 10), which supports their closer evolutionary relationship (Almeida et al., 2023). This configuration likely enhances coxal stability. In contrast, the restricted origin in other bees and apoid wasps (char: 14, state: 1; Figs. 5A, 10) may reflect adaptations for less dynamic flight patterns, as seen in Megachilidae, which prioritize load-carrying for nest provisioning (Michener, 2007). Similarly, **IIIvolm** (M. metafurca-abdominosternalis lateralis) shows an anterior origin in Apidae and *O*. *flavescens* (char: 17, state: 0; Figs. 6B, 9E, 10), potentially improving metasomal flexibility for pollen transport or nest manipulation, contrasting with the posterior origin in other taxa (char: 17, state: 1; Figs. 9F, 10).

The muscle **IIpcm4** (M. propleuro-coxalis posterior) exhibits a branched origin in some lineages (char: 10, state: 0; Figs. 8E, 10), transitioning from fan-shaped to V-shaped forms, as noted by Wille (1956). This branching likely increases muscle force distribution, supporting the powerful leg movements required for digging in ground-nesting bees, like Andrenidae or Halictidae (Michener, 2007). The not-branched form (char: 10, state: 1; Figs. 4D, 10) in other lineages may favor streamlined leg motion (Dickinson, 2006), suitable for hovering or precise landing. Changes of **IIpcm4** in unrelated bee lineages suggest that biomechanical demands may drive morphological variation even more than the degree of phylogenetic proximity.

The absence of separation between **IItpm7b** and **IItpm7c** by the mesepisternal ridge in bees (char: 8, state: 1; Figs. 4A, 8C, 10) is a potential synapomorphy, distinguishing them from most apoid wasps. This fused configuration may enhance wing flexor efficiency, supporting sustained flight (Dudley, 2000), as observed in behaviors as hovering and foraging on flowers. In contrast, the separated configuration in apoid wasps (char: 8, state: 0; Figs. 8B, 10) likely may reflects their predatory lifestyle, requiring rapid and powerful wing movements (Chapman, 2013).

Several autapomorphies have been identified within specific taxa. In *A. mellifera*, unique features include the lateral origin of the muscle group **Idvm18** (char: 5, state: 0; Figs. 7A, 10), the restricted origin of the muscle group **IIspm1** to the anterior area of the mesepisternum (char: 9, state: 0; Figs. 8C, 10) and the medial insertion of **IIIdlm3** (char: 7, state: 0; Figs. 7E, 10). In *L. huberi*, the extension of the origin of **Iscm5** to the free basal portion of the mesofurcal arms (char: 6, state: 1; Figs. 8A, 10) highlight species-specific adaptations. The restricted origin point of **IIscm6** (char: 12, state: 0; Figs. 4C, 10) in *M*. *quadrifasciata* is suggested to be another autapomorphy. Such autapomorphies underscore the importance of detailed analyses of the skeletomusculature for understanding evolutionary differentiation, as emphasized by Vilhelmsen, Mikó & Krogmann (2010).

These morpho-functional patterns suggest that mesosomal musculature variations are not randomly distributed, but may have been shaped by ecological and biomechanical demands. For example, muscle configurations supporting head mobility (**Idlm1**, **Ivlm3**) may enhance foraging efficiency in diverse habitats, while those affecting coxal and metasomal movement (**IIIscm2**, **IIIvolm**) are likely to improve flight and nest-building behaviors. Moreover, potential correlations exist among these variations; for instance, shifts in head depressor muscles (*e.g.*, Ivlm3) could interact with coxal remotor configurations (*e.g.*, IIIscm2) to optimize overall body stability during hovering or burrowing, reflecting integrated adaptations across the mesosoma. While our descriptive focus highlights these patterns, a more synthetic analysis, integrating quantitative metrics of muscle force, attachment geometry, and behavioral data, could reveal deeper functional interdependencies. Future studies integrating biomechanical modeling and kinematic analyses could further elucidate how these variations influence flight dynamics, pollen collection, or nest construction, serving as a logical extension of the morphological framework established here.

## Conclusion

This study identified distinct patterns of variation within a largely conserved anatomical framework of the mesosomal musculature in bees by examining 10 bee species and three apoid wasp taxa, while also exploring the phylogenetic importance of this variation. All 58 muscle groups were consistently present across the examined taxa, with no absences noted. Despite body-size differences, the observed variations were not directly attributable to size but rather to phylogenetic and potential biomechanical factors, as discussed. Of the 58 muscle groups analyzed, only 16 exhibit significant variation, primarily in origin points, with the prothoracic (*e.g.*, **Idlm1**, **Ivlm3**) and metathoracic (*e.g.*, **IIIscm2**, **IIIvolm**) muscles proving most informative, likely due to greater functional constraints on the mesothorax, which houses the primary flight engine (indirect flight muscles) and thus exhibits higher conservation. Some synapomorphies, such as the dorsomedial **Idlm1** in Meliponini and the extended **IIIscm2** in Andrenidae, Colletidae, and Halictidae, underscore close phylogenetic relationships within these lineages. In contrast, the fused **IItpm7b** and **IItpm7c** of bees distinguish them from their close relatives in the Apoidea. These variations suggest biomechanical adaptations for flight, foraging, and nesting, as required by ground-nesting bees, highlighting functional specialization. The detailed character systems established here provide a foundation for phylogenetic interpretations of the variation in the mesosomal musculature of bees. Expanding taxonomic sampling in the future will likely enhance our understanding of mesosomal evolution and its impact on the diverse ecological strategies that contribute to the evolutionary success of bees and apoid wasps. Moreover, future research employing advanced techniques, such as micro-computed tomography, will undoubtedly help improve our knowledge of muscular variation in Apoidea.

##  Supplemental Information

10.7717/peerj.20532/supp-1 Supplemental Information 1 Terminology applied to the mesosomal skeletomusculature and variation Tabulation of the terminology applied to the mesosomal skeletomusculature used in this work and contrasted with Snodgrass (1942), Vilhelmsen (2000a); Vilhelmsen (2000b), Mikó et al. (2007), Friedrich & Beutel (2008), Vilhelmsen, Mikó & Krogmann (2010), Willsch et al. (2020), Aibekova et al. (2022), and Lieberman et al. (2022). List of muscle groups with variation detected within bees and Apoidea.

10.7717/peerj.20532/supp-2 Supplemental Information 2 Character matrix 17 morphological characters and their respective states derived from the extrinsic mesosomal musculature of bees, which were scored for the 13 species of Apoidea (ten bee and three wasp species).

## Additional Information and Declarations

### Data Availability

The following information was supplied regarding data availability: The raw data is available in the Supplemental Files.
